# Empirical Research on the Critical Success Factors of Construction Program

**DOI:** 10.1155/2022/9701963

**Published:** 2022-05-27

**Authors:** Chunmei Zhou, Zheng He, Ping Hu, Hongyan Yan

**Affiliations:** Department of Construction Management, Hunan University of Finance and Economics, Changsha 410205, Hunan, China

## Abstract

Scientific identification of the factors that underpin the success of construction program can effectively promote the success of construction program. Based on literature statistics, this paper developed the measurement items of key success factors of construction program in Chinese context. Based on exploratory factor analysis, seven key success factors of construction program were extracted. Then, the excellence model of construction program was constructed by referring to the ideas, concepts, and theoretical mechanisms of EFQM model. The research conclusions of this paper provide a systematic and holistic guidance for the successful implementation of the program by the construction program organization and promote the success of the program.

## 1. Introduction

With the economic development having been switched from high-speed development to high-quality development, China is now facing a critical period of development mode transformation, economic structure optimization, and growth momentum conversion. Against this background, the construction industry needs to achieve high-quality development through transformation and upgrading. With the continuous expansion of the scale of the construction industry and the implementation of national strategies such as “Belt and Road Initiative” and “New Urbanization,” the number and scale of construction programs are increasing day by day, and the construction program aims to pursue higher values and benefits and achieve the expected goals through unified coordination and management of multiple projects so as to promote the high-quality development of the construction industry. Compared with an individual project, the construction program is not only larger in scale, but also characterized with higher complexity, longer duration, higher cost, and greater risk, and it also has more significant social and economic impact [1]. Therefore, it is of particular importance to think about how to promote the success of the construction program so as to facilitate the upgrading and transformation of the construction industry and further enhance the competitiveness of the industry. The key success factors of the construction program are the factors that are managed and influenced by the organization in an effort to promote the success of the program. Clarifying the key success factors of the construction program can help managers to figure out the management priorities and difficulties and allocate resources rationally to achieve the purpose of promoting the success of the program [2].

At present, the academic circles in foreign countries have conducted a large number of theoretical and empirical studies on the key success factors of the program, and the scholars have set forth different views on this matter [3,4]. With the development of the theory of construction program management, the research on the key success factors of construction program has gradually developed. Scholars such as Haadir et al. [5] (2011) believe that good communication, support from senior executives, monitoring and feedback, correct project goals, teamwork, power decentralization, and adequate financial support are the key factors to ensure the success of the construction program. Scholars such as Patanakul et al. [6] (2016) put forward six key factors to promote the success of construction programs, namely, the pursuit of nonfinancial target benefits, product service life, multiple stakeholders, complexity, political environment, and compliance with mandatory project management procedures; scholars such as Gamil et al. [7] (2017) believe that the establishment of an appropriate certification system for contractors and consulting agencies, reasonable cost and time planning, effective communication and coordination platforms, and advanced technologies are the key factors for the success of the construction program; Shao et al. [8] (2018) believe that, apart from adequate financial support, delivery capability, organizational capability, innovation capability, organizational adaptability, project flexibility, and organizational stability are also key success factors for the program.

The research results mentioned above have enriched the theoretical system of the key success factors of the construction program and formed a general key success factor model of the construction program. However, the researchers have not reached a unified conclusion as to the key factors that influence the construction program. At the same time, cultural and industrial discrepancy may affect the applicability of the key success factor frameworks of construction program proposed by different scholars (Shao et al., 2012 [9]), and Pellegrinelli et al. [10] (2007) also emphasized the importance of environment for program management. Therefore, it is necessary to study the key success factors of construction program in our country based on the Chinese context. Based on literature statistics and exploratory factor analysis, this paper refines the key success factors of the construction program and builds an excellence model of the construction program by referring to the ideas, concepts, and theoretical mechanism of the EFQM model. The research in this article provides a new framework system of critical success factors for construction program managers and helps them to quickly clarify the dimensions of project management, improve management levels, define development directions, and promote the success of construction programs.

## 2. Definition of Success Criteria for Construction Programs

There is a causal relationship between the success criteria of the construction program and the key success factors, and different key success factors will be identified for different success criteria. Therefore, defining the meaning and the success criteria of the construction program is the basis for scientifically identifying the key success factors of the program. Based on the previous research, this paper develops the standard measurement items for the success of construction programs in the Chinese context based on literature statistics and China's construction program management practices. On the basis of the data collected by the questionnaire survey, the SPSS22.0 statistical analysis software is adopted as an auxiliary tool to carry out exploratory factor analysis for the success criteria of the construction program, and the success criteria of the construction program are extracted: the success of the construction program management, the success of the construction program organization strategy, and the harmony of the stakeholders of the construction program.

## 3. Theoretical Construction of Key Success Factors of Construction Program

At present, the research on the key success factors of construction program is still in its infancy worldwide, and there are few related documents. Seven papers on the key success factors of construction program published in international authoritative journals were selected. By comparing and analyzing the key success factors between construction projects and construction programs, the results indicate that more emphasis is put on the key influence of “executive support,” “clear goals,” and other factors on the success of the construction program, while more emphasis is put on the key influence of factors such as “team member ability” and “team communication” and other factors on the success of the construction project.

Literature analysis lays the foundation for identifying the key success factors of construction program. Due to the differences in the construction environment of the programs in various countries, this paper draws on the statistical conclusions of literature, the 66 key success factors of construction program proposed by scholars such as Sarmad Kiania [11], and the in-depth interviews carried out with university scholars, program managers, program executives, and other experts to explore the key factors affecting the success of the construction program in China. Also, this paper supplements and discovers the hidden factors of the construction program and summarizes the measurement items for the key success factors of the construction program in the Chinese context, as shown in Table 1.

## 4. Empirical Analysis of Key Success Factors of Construction Program Based on Exploratory Factor Analysis

This paper adopts questionnaire survey to carry out empirical research on the key success factors of construction program, and a total of 32 valid questionnaires were collected. The subjects of the survey are company executives, program managers, program management support personnel/auxiliary personnel, etc. Most of the subjects are highly educated and have relatively rich work experience, so the questionnaire results are representative, reliable, and authentic.

### 4.1. Adaptability Test of Factors

In order to analyze the quality of the questionnaires, this paper needs to test the reliability and validity of the scale data. The output results suggest that the correlation coefficient matrix is “nonpositive definite matrix.” Neither KMO nor Bartlett's test shows the results. The main reasons mainly come from two aspects: (1) The sample size is too small while there are too many indicators. (2) The correlation between certain variables is too strong. There are 40 measurement items for the key success factors of the construction program, but only 32 questionnaires were effectively collected. The main reason is that the management idea of the construction program has not been effectively promoted in China's program practice. Therefore, there is a need to process the data in order to analyze the key success factors of construction program. The main measure taken is to reduce the number of measurement items. Through the analysis of the correlation of indicators, eleven items were deleted from the key success factor measurement items of the construction program, so as to meet the requirements of the quality inspection of the factor analysis, as shown in Table 2.

### 4.2. Structural Factor Variant

SPSS 22.0 tool was used to perform exploratory factor analysis on the scale data, and the common factor is extracted based on the standard where the feature value is greater than 1 and the cumulative contribution rate is greater than 80%, and the factor analysis results are shown in Table 3.

From the results in Table 3, it can be seen that the cumulative variance contribution rate explained by the first 6 common factors is 80.583%, indicating that these 6 factors have integrated 80.583% of the information of original 29 success factors from the key success factors of the construction program, which can basically reflect the essential information of the sample.

### 4.3. Factor Variable Explanation

In order to further explain the 6 common factors that have been extracted, the maximum variance orthogonal rotation method is used to perform factor rotation on the extracted factors. The rotated factor loading matrix is shown in Table 4. The value in the factor loading matrix reflects the importance of the corresponding key success factor index of the construction program to each factor.

Factor 1 explains the measurement items of SF11, SF36, SF6, SF4, and SF5 and reflects that the completeness of the construction program organization and management system directly affects the success or failure of the construction program, so it is named the organization/management standard system completeness factor.

Factor 2 explains the measurement items of SF27, SF13, SF26, SF29, SF25, and SF37, reflects the connotation of the whole process management of the construction program, and shows that the effective management of the whole process of the construction program is the key factor to promote the success of the program, so it is named excellent program management factor.

Factor 3 explains the measurement items of SF34, SF20, SF19, SF33, SF38, and SF14 and reflects that high competency of the construction program management team can effectively promote the success of the program. Therefore, it is named the excellent program management team factor.

Factor 4 explains the measurement items of SF18, SF40, SF12, SF3, SF39, and SF1. It is divided into two factors since it cannot effectively explain the key success factors of specific construction programs. SF18, SF12, and SF1 explain the organization strategy and goal of construction programs, indicating that organizational strategy has a guiding role in the implementation of the program. Organizational strategy is the basis for the success of the program, so it is named the organizational strategy/clear target factor; SF40 and SF39 explain the role of government support in promoting the success of the construction program, and it is named the government support factor.

Factor 5 explains the measurement items of SF16, SF31, and SF30 and reflects the interest demands and cooperation of the stakeholders of the construction program, so it is named the stakeholder cooperation factor.

Factor 6 explains the measurement items of SF7 and SF8, indicating that the support of senior executives is inseparable from the success of the program, and it is named the senior executive support factor.

### 4.4. Results and Discussion

#### 4.4.1. Organizational Strategy/Clear Target Factor

Organizational strategy and goals are the blueprint and outlook for the strategic goals and mission scope of the construction program. The implementation of any project/program is inseparable from a clear organizational strategy and target.

The achievability of the project objectives is the prerequisite for the smooth implementation of a construction project. Researches have shown that the formulation of an implementable plan can substantively improve the possibility of achieving overall success and project progress, thereby improving project performance [12]. Therefore, it is the initial and prior task to formulate reasonable and implementable strategic goals when building the construction program, and clarifying the project/Program targets will help the stakeholders of the construction program to figure out their responsibilities and facilitate the smooth implementation of the project/program. Researchers, such as Shenhar et al. [13] (2001), believe that the organization's strategy and project targets should be well-aligned to ensure their complementarity and directional consistency so as to promote the success of the construction program.

#### 4.4.2. Senior Executives Support Factor

Senior executive support refers to the phenomenon that the senior executives provide various resources as well as written and verbal support to the program team during the whole process of building the construction program. Existing studies believe that senior executive support is one of the necessary conditions for project success (Crosby [14], 2012). Senior executives are the core personnel of project/program and the executive to accomplish organizational strategy and target; through the tactic/strategic support of the senior executives to the project/program, the project/program management process can be improved, which can have a positive impact on the project and program objectives and thereby improve the performance of the program (Duan Zhicheng [15], 2013).

Senior executive support promotes the success of the construction program mainly through the following aspects: The senior executives promise to complete the program; pay attention to the progress of the construction program; actively participate in the program and provide necessary support; ensure that the project/program has an appropriate priority.

#### 4.4.3. Completeness Factor of Organization/Management and Other Normative Systems

Standardization of the organization and management system is the basic element for the success of construction projects/programs. By constructing a positive organization culture and establishing a reasonable organization structure for the construction program, the ability of the organization and individuals can be effectively boosted and the effectiveness of the policy and strategic goals can be improved, so that the management efficiency of construction project/program can be raised to promote the success of the program [15].

The organization of the construction program is determined by the strategic goal of the program, and the organization is an important foundation and support for the realization of the strategic target. Construction program management is a highly integrated management, and the program organization should also achieve the unity of powers, responsibilities, and benefits to ensure that the organization can effectively perform its functions. The larger the construction program and the more complex the task, the more it is necessary to build an effective organization and adopt the correct work process to comprehensively consider the program's targets and resources, so that the program management can be well-organized and the program's strategic targets can be achieved. The positive culture of the construction program has an important impact on the employees participating in the construction program and the program itself (Zuo, Zillante [16], 2005). At the same time, the project/program organizational culture can promote the governance of the project/program, boost employees' work enthusiasm, reduce contradictions and conflicts, and help to harmonize the atmosphere among organizations (Yang et al. [17], 2018).

#### 4.4.4. Excellent Program Management Team Factor

The construction program management team includes the team members with the construction program managers as the core, the management technology used by the managers/team, the application of capabilities, and the needs of the managers/team. Scholars such as Belassi et al. (1996) [18] believe that the management skill of the project manager department is the most important key factor for project success; scholars such as Kog et al. (1999) [19] found that the key factors affecting project success include the project manager's emphasis on the project and the experience of the project manager, etc. Therefore, it is helpful to select an experienced and capable construction program/project manager to improve the organization and individual capabilities and formulate effective strategies and integrate internal and external resources, so as to improve the efficiency of the construction program/project management and effectively promote the success of the program/project [15].

As the core team of the whole process management, the construction program management team plays an important role in dealing with some complex situations and systems and ensuring the implementation of the construction program and the successful delivery of the program. The construction program management office is a project management organization specially set up for project management, which influences the success of the program by providing practical methods for construction program management. The ability of the members of the construction program management team is matched with the application of technology; that is, the stronger the ability of the program team members, the higher the technical requirements, which is conducive to the success of the project. The manager of the construction program can also resolve the errors and conflicts of a certain subproject or unit by allocating resources to ensure the realization of the goal of the construction program.

#### 4.4.5. Excellent Program Management Factor

According to project management theory, project management ability is the key to ensuring project value addition. Construction program management is the key stage for the completion of the construction program products, which must rely on scientific and effective management methods to grasp the critical points of implementation so as to promote the success of the program. Research results show that 62.4% of experienced project/program managers believe that when the project/program management is successful, the project/program will generally succeed [20]. In this sense, effective program management is the foundation of the success of program [21].

The implementation of the construction program involves the scope, time, cost, quality, human resources, communication, resources, information and risk, and other management fields of the program. In light of the traditional “iron triangle-time management, cost management, quality management”, etc., it is necessary to take effective control measures under target constraints. At the same time, control measures shall also be strengthened in management areas such as resource management, information management, and risk management.

#### 4.4.6. Stakeholder Cooperation Factor

The needs, expectations, requirements, and behaviors of the stakeholders of the construction program can impose influence on or are affected by the program to varying degrees. Their duty-performing ability and level of performance are directly related to the success or failure of the program. The satisfaction of the different interest needs of the stakeholders of the construction project/program and the harmonious relationship between the stakeholders, which benefit all participants, are important factors for the success of the project group; and the inability to achieve effective cooperation among the stakeholders of the project/program will cause organizational chaos [22] and may lead to project/program failure.

Strengthening the communication, coordination, and cooperation among the stakeholders of the construction program\project is essential to the stakeholder relationship management of the construction program. It can promote timely communication, reduce adverse effects, avoid conflicts between departments, and resolve incidents that hinder progress and can help to create a coordinated and harmonious environment and improve the management level and operational efficiency of the entire program, thereby promoting the success of the program.

#### 4.4.7. Government Support Factor

In the decision-making and construction of major construction projects/program, the role of the government is irreplaceable, and the relationship between the government and the construction program is inseparable.

Program construction is a large-scale project which entails a lot of attention from the government, and governmental approval is required for various processes involved during the construction process of the program. At the same time, the government also plays a role in the supervision and management of the program construction. Studies have found that the government, which supervises and regulates the implementation process of the construction program through supervisory and regulatory institutions, can achieve the purpose of preventing corruption and promoting the program performance. Efficient public institutions have a great impact on the success of the project [23], and the higher the efficiency of the government, the faster the construction of the project/program, and the more likely the project/program will be successful. By providing effective guarantees and policy support, the government can promote the success of the program [24]. The support of national policies greatly encourages the government executives to support the construction of the program. The government executives effectively promote the performance of the program by facilitating the project's preliminary consultation and approval process. Government credit is equally important to the success of the project, and the trustworthiness of all stakeholders in the project/program is vital to the continuation of cooperation, and any party's breach of contract may lead to the termination of the project/program.

## 5. Excellence Model of Construction Program

The EFQM model is the first excellence model selected by the European Foundation for Quality Management through evaluation. The EFQM model measures project performance and shows the ideal strategy and targets of the project to elevate its performance to the highest level (Wongrassamee et al. [25], 2003). Although the analytical methods used by the EFQM model are vastly different from those used in the field of engineering project management, it is found that that there have been research results achieved by applying the EFQM model in construction field [26] according to papers published in this field. The EFQM model embodies the key influence between the facilitation factors (process) and organizational performance (results). Therefore, this paper attempts to introduce the EFQM model into the mechanism of interaction between the key success factors and the success criteria of the construction program to build an excellence model for the construction program.

This paper refers to the thought of Westerveld [27] (2003) to apply the EFQM model in the project success field. Its hypothesis is to divide project management into two fields, namely, the result field and the facilitation field. The result field represents the project success criterion and the facilitation field represents the key success factors. In this light, this paper interprets the key success factors of the construction program as the facilitation field and the success criteria of the construction program as the result field.

In the facilitation field of the excellence model of the construction program, leadership is a behavior in which managers in an organization use their power to exert influence on their subordinates in order to achieve the goals of the organization. Senior executive support refers to the support of senior executives to their subordinates, and leadership can be interpreted as senior executive support; the personnel, policies and strategies, partners, and resources covered by the facilitation field can be, respectively, interpreted as excellent program management team, clear strategies/targets, complete organization/management, and stakeholder cooperation and government support; the process reflects the whole process of constructing the program, and therefore the process can be deemed as excellent program management. In the result field of the construction program excellence model, the dimensions of personnel results and customer satisfaction correspond to the harmony (satisfaction) of stakeholders; social influence reflects the long-term performance of the organization, that is, the success of the organization's strategy; corporate performance is interpreted by the success of project management.

**Figure 1:**
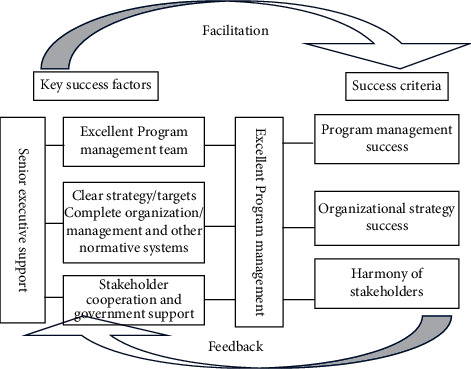
Excellence model of construction program.

Based on the above analysis, this paper constructs an excellence model of the construction program, which reveals the relationship between the success of the program and the key success factors as well as the enabling paths, as shown in Figure 1.

The excellence model of the construction program based on the EFQM model organically combines the “success criteria of construction program” in the success result field and “key success factors of construction program” in the facilitation field. The two constitute a facilitation-feedback relationship; that is, the key success factors of the construction program facilitate the success of the program while the success of the program reacts upon the whole process management of the program. The scientific definition of the success criteria of the construction program plays a guiding role in defining the key success factors of the construction program managers in the whole process management of the program and helps the managers to judge and effectively control the key success factors.

## 6. Conclusion

Key success factors of the program play a decisive role in determining the success of the construction program. Therefore, in-depth and systematic research on the key success factors of the program will help the construction program organization to focus on the investment in key factors and increase the likelihood of program success.

Based on literature statistics, this paper explores the key factors affecting the success of construction programs in China, supplements and excavates the hidden factors of the construction program, and develops the measurement items for the key success factors of the construction program in the Chinese context. Using the data collected by the questionnaire survey, SPSS22.0 statistical analysis software is used as an auxiliary tool for exploratory factor analysis of the key success factors of the construction program, and the key success factors of the construction program are refined: Key success factors in seven dimensions, including clear organizational strategy/targets, senior executive support, government support, organization/management and other standard systems, excellent program management team, stakeholder cooperation, and excellent program management. The ideas, concepts, and theoretical mechanisms of the EFQM model construction program are drawn on to organically integrate the “success criteria of construction program” in the result field and “key success factors of the construction program” in the facilitation field to construct an excellence model of the construction program.

The key success factors of the construction program and the excellence model of the construction program established in this paper provide a directional guide for the systematic and holistic success of the implementation of the program by the construction program organization. Real-time monitoring and process control are carried out during the implementation of the program to make the implementation of the program more flexible, reduce the risk of the program, and promote the success of the program.

## Figures and Tables

**Table 1:** Measurement items of key success factors of construction program.

Key success factors measurement items of construction program
SF1. Clear vision, strategic goals, and mission.
SF2. Well-defined and realistic organizational goals.
SF3. Well aware of opportunities and threats for industry development.
SF4. Have a positive organizational culture and organizational structure system.
SF5. Have a clear and documented accountability system.
SF6. Have a good communication and management system.
SF7. Strategic support from executives.
SF8. The senior executives fully trust and authorize the program/subproject management team.
SF9. The strategic alignment between the objectives of the program and the technology.
SF10. Correct feasibility study.
SF11. Reasonable program financing method.
SF12. Reasonable program delivery system.
SF13. Allocate correct and sufficient resources to each subproject such as funds, materials, and equipment.
SF14. Stable employment of the employee system.
SF15. Regular evaluation of the objectives of the program and subprojects.
SF16. Adequate communication among program stakeholders.
SF17. Clear program objectives, deliverables, and benefits.
SF18. The consistency of the program objectives and organizational strategy.
SF19. Outstanding program/project manager.
SF20. Familiar with the exact information needs of executives.
SF21. Correct program cost estimation.
SF22. Appropriate program budget allocation.
SF23. Effective program cost management.
SF24. Reasonable program/subproject schedule.
SF25. Effective program/subproject schedule management.
SF26. Effective program quality management.
SF27. Effective program change management.
SF28. Effective communication management to control disputes and conflicts.
SF29. Effective risk management.
SF30. Effective stakeholder management.
SF31. Reasonable distribution of benefits among stakeholders.
SF32. Reasonably adjust and guide the program/subproject performance based on the strategic objectives.
SF33. The program manager pays attention to the mutual influence between the program/subproject targets.
SF34. Use mature tools, techniques, and management processes in program management.
SF35. Use integrated and coordinated management to achieve strategic goals.
SF36. There are enough program management business cases/plan charters for reference.
SF37. Provide continuous financial support according to the program budget.
SF38. Powerful and integrated program management office.
SF39. The government's policy affirmation and support for the program.
SF40. Government departments provide efficient services for the program implementation.

**Table 2:** KMO and Bartlett's validity test.

Kaiser–Meyer–Olkin	Questionnaire adequacy measurement	0.645
Bartlett's sphericity test	*χ* ^2^	1035.466
	Df (degree of freedom)	406
	Sig. (significance level)	0.000

**Table 3:** Initial and rotated factor variation contribution rate of indicators.

Factors	Initial eigenvalue			Rotated sum of squares loaded			Rotated sum of squares loaded	
	Total (%)	Variance (%)	Cumulative (%)	Total (%)	Variance (%)	Cumulative (%)	Total (%)	Variance (%)
1	15.985	55.121	55.121	15.985	55.121	55.121	4.875	16.810
2	1.896	6.536	61.657	1.896	6.536	61.657	4.390	15.139
3	1.587	5.474	67.131	1.587	5.474	67.131	4.255	14.673
4	1.431	4.934	72.064	1.431	4.934	72.064	4.101	14.143
5	1.382	4.765	76.829	1.382	4.765	76.829	3.173	10.943
6	1.089	3.754	80.583	1.089	3.754	80.583	2.574	8.876
…								
29	0.001	0.003	100.000					

**Table 4:** Rotated factor loading matrix of key success factors of construction program.

Factor 1	
Component	Factor loading
SF11	0.876
SF36	0.800
SF6	0.622
SF4	0.594
SF5	0.576
Factor 2	
SF27	0.825
SF13	0.662
SF26	0.653
SF29	0.620
SF25	0.612
SF37	0.609
SF23	0.540
Factor 3	
SF34	0.824
SF20	0.777
SF19	0.669
SF33	0.615
SF38	0.589
SF14	0.443
Factor 4	
Component	Factor loading
SF18	0.791
SF40	0.726
SF12	0.615
SF3	0.586
SF39	0.555
SF1	0.465
Factor 5	
SF16	0.854
SF31	0.640
SF30	0.623
Factor 6	
SF7	0.781
SF8	0.577

## Data Availability

All data are included in the manuscript.
